# Neurophysiological validation of simultaneous intrinsic and reflexive joint impedance estimates

**DOI:** 10.1186/s12984-021-00809-3

**Published:** 2021-02-17

**Authors:** Ronald C. van ’t Veld, Alfred C. Schouten, Herman van der Kooij, Edwin H. F. van Asseldonk

**Affiliations:** 1 Department of Biomechanical Engineering, University of Twente, Enschede, The Netherlands; 2 Department of Biomechanical Engineering, Delft University of Technology, Delft, The Netherlands

**Keywords:** Joint resistance, System identification, Parallel-cascade model, Electromyography, Validation

## Abstract

**Background:**

People with brain or neural injuries, such as cerebral palsy or spinal cord injury, commonly have joint hyper-resistance. Diagnosis and treatment of joint hyper-resistance is challenging due to a mix of tonic and phasic contributions. The parallel-cascade (PC) system identification technique offers a potential solution to disentangle the intrinsic (tonic) and reflexive (phasic) contributions to joint impedance, i.e. resistance. However, a simultaneous neurophysiological validation of both intrinsic and reflexive joint impedances is lacking. This simultaneous validation is important given the mix of tonic and phasic contributions to joint hyper-resistance. Therefore, the main goal of this paper is to perform a group-level neurophysiological validation of the PC system identification technique using electromyography (EMG) measurements.

**Methods:**

Ten healthy people participated in the study. Perturbations were applied to the ankle joint to elicit reflexes and allow for system identification. Participants completed 20 hold periods of 60 seconds, assumed to have constant joint impedance, with varying magnitudes of intrinsic and reflexive joint impedances across periods. Each hold period provided a paired data point between the PC-based estimates and neurophysiological measures, i.e. between intrinsic stiffness and background EMG, and between reflexive gain and reflex EMG.

**Results:**

The intrinsic paired data points, with all subjects combined, were strongly correlated, with a range of $r = [0.87\ 0.91]$ in both ankle plantarflexors and dorsiflexors. The reflexive paired data points were moderately correlated, with $r = [0.64\ 0.69]$ in the ankle plantarflexors only.

**Conclusion:**

An agreement with the neurophysiological basis on which PC algorithms are built is necessary to support its clinical application in people with joint hyper-resistance. Our results show this agreement for the PC system identification technique on group-level. Consequently, these results show the validity of the use of the technique for the integrated assessment and training of people with joint hyper-resistance in clinical practice.

## Background

People with brain or neural injuries, such as cerebral palsy or spinal cord injury, commonly have an increased joint resistance (or ’hyper-resistance’) [1]. This joint hyper-resistance can severely impair both walking ability and functional independence. The origin of the hyper-resistance can vary and arises from one or multiple of the following categories [2]:

- **Intrinsic:**
  1. a tissue-related non-neural origin, e.g. shortened tissue or fibrosis;
  2. a tonic neural origin, i.e. involuntary background muscle activation;

- **Reflexive:**
  3 a phasic neural origin, i.e. stretch hyperreflexia (’spasticity’).

The mixed origin of the joint hyper-resistance creates a challenge in the diagnosis and treatment of hyper-resistance. Ideally, diagnostic methods unravel the three contributions to hyper-resistance [2]. However, current clinical practise lacks a valid and reliable procedure to unravel these contributions. Besides, current treatment includes non-specific interventions with questionable cost-effectiveness. For example, Botulinum neurotoxin injections reduce both involuntary background activation and spasticity, but also the ability to perform voluntary muscle contractions [3, 4].

**Fig. 1:**
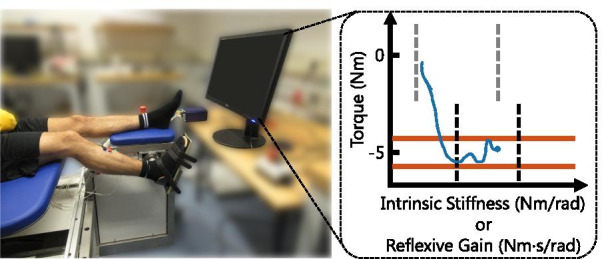
Experimental setup overview. Participants were seated on an adjustable chair with their right foot connected to an actuator, applying perturbations around the ankle joint. Feedback was given using a (blue) 2D trace on both torque (y-axis) and an impedance parameter (x-axis). On the y-axis, a (red) torque target was shown around either 0 or −5 Nm. On the x-axis, (black-dashed) reference lines were shown with the average magnitude of the impedance parameter from previously completed 60 s hold periods at each torque level. In the specific example situation depicted, a participant would have had the following two tasks: (1) (y-axis) keep voluntary torque stable within the target boundaries around −5 Nm; and (2) (x-axis) keep the impedance parameter stable and away from the black-dashed reference lines shown

The parallel-cascade (PC) system identification technique offers a potential solution for the integrated assessment and treatment of joint hyper-resistance [5]. The technique disentangles and simultaneously estimates the intrinsic and reflexive contributions to joint impedance, i.e. the joint’s resistance to imposed motion. PC algorithms have been used successfully to assess joint hyper-resistance compared to a control group and to assess the effect of treatments on joint resistance [6, 7]. Moreover, an online PC algorithm is available, which directly estimates joint impedance contributions during data acquisition and can consequently be used to provide biofeedback [8]. Training people using this joint impedance biofeedback was shown to achieve voluntary within-session modulation of both intrinsic and reflexive contributions independently [9]. This ability to train joint impedance modulation enables a novel treatment using the PC algorithm to specifically reduce spasticity. For such a treatment, the within-session modulation of reflexive impedance should be consolidated to an across-session, long-term effect. This transformation from within- to across-session effects are key for an effective intervention and has been shown in electromyography (EMG)-based operant conditioning protocols [10, 11].

The main goal of this paper is to perform a group-level neurophysiological validation of the PC system identification technique to support its clinical application. The validation is performed using the online PC algorithm, because of the ability to provide biofeedback. Primarily, the neurophysiological validation will be performed by investigating the linear association of the system identification outcome measures [9] with equivalent EMG-based outcome measures [10, 11]. We expect the following parameters to be correlated (also see pilot experiment [12]):

1. estimated intrinsic joint stiffness is correlated with background EMG activity in both ankle plantarflexors and dorsiflexors [13];
2. estimated reflexive gain is correlated with reflex EMG activity in the ankle plantarflexors only [9].

Secondarily, the effect of varying voluntary torque on these linear associations is investigated, as the various assessment and treatment methodologies use a mix of relaxed and tonically activated plantarflexors [6, 11]. The change between the relaxed and activated conditions is known to influence both the intrinsic joint stiffness and reflexive gain [14].

This study investigates the agreement between the PC system identification technique and the neurophysiological basis on which it is built [5]. The association between PC algorithms and EMG-based outcome measures has been investigated for reflexive contributions only [9, 15, 16]. However, validating both intrinsic and reflexive pathways simultaneously is important, given the mixed intrinsic and reflexive origins of joint hyper-resistance. Besides, all previous results investigating this linear association were restricted by limited or no variation in voluntary muscle activation. A successful validation would increase clinical confidence in the PC technology when used for people with joint hyper-resistance.

## Methods

### Participants

Ten people with no history of neuromuscular disorders participated in the study (4 female, age 27.8±1.7 yr). The EEMCS/ET ethics committee of the University of Twente approved the study and all participants provided written informed consent.

### Apparatus

The experiment was executed using an adjustable chair, actuator, EMG device and feedback screen, see Fig. 1. Participants were seated on the adjustable chair, which supported the upper leg and upper body, while controlling for hip (120$^{\circ }$) and knee (150$^{\circ }$) angles. The right foot was connected to the actuator, integrated into the frame of the chair, using a rigid footplate and Velcro straps. The ankle and actuator axes of rotation were visually aligned before the start of the experiment, minimizing knee translation due to the applied ankle rotations.

A one degree of freedom actuator (Moog, Nieuw-Vennep, The Netherlands) was used to apply the perturbations required for the PC algorithm. These position perturbations were applied in the sagittal plane around the ankle joint. The actuator’s encoder measured the position and velocity of the footplate as indirect measure of the imposed ankle position (i.e. angle) and velocity (i.e. angular velocity). Similarly, a torque sensor was placed between the footplate and actuator as indirect measure of resulting ankle torque. Position, velocity and torque were recorded at 2048 Hz, all defined positive in dorsiflexion direction.

A Porti EMG device (TMSi, Oldenzaal, the Netherlands) recorded activity of the Soleus (SOL), Tibialis Anterior (TA), Gastrocnemius Medialis and Lateralis (GM and GL) muscles at 2048 Hz. EMG electrodes were placed according to the SENIAM guidelines [17].

A feedback screen provided biofeedback at a rate around 25 Hz using Matlab 2017b (Mathworks, Natick, MA, USA). The 2D feedback screen, see Fig. 1, visualized a 6 s historic trace of the low-pass filtered torque (2nd-order, 0.1 Hz, critically-damped) in combination with the intrinsic stiffness or reflexive gain parameter from the online PC algorithm. Using the online PC algorithm estimates was challenging, as these estimates had a long transient period of about 15 s before becoming reliable [8, 9]. Therefore, each data collection period only started when both researcher and participant mutually agreed that the participant could keep the feedback constant.

### Experimental protocol

First, an appropriate joint angle to elicit reflexes was determined for each participant, as reflexes depend on joint angle [14]. An initial trial was run at a 90$^{\circ }$ ankle angle, i.e. the angle between shank and foot determined using a goniometer. This initial trial also familiarized participants with the robotic setup, applied perturbations, feedback screen and task instructions. If participants had an estimated reflexive gain below 3 Nm$\cdot$s/rad at 0 Nm torque, the ankle angle was increased in 5$^{\circ }$ steps. This more dorsiflexed ankle angle was used to increase the minimum reflex magnitudes and avoid multiple measurements close to zero distorting data analysis. Eventually, 5 participants performed the experiment at a 90$^{\circ }$ ankle angle and another 5 at 95$^{\circ }$.

The experiment was split in 4 blocks of max. 15 min with continuous perturbations and biofeedback. A 3−5 min break was included between blocks to avoid fatigue and loss of concentration. Participants were instructed to keep their voluntary torque between the two torque target boundaries. Moreover, participants were instructed to generate this torque by focusing on ankle rotation without using the upper leg. The torque target switched between the 0 and −5 Nm levels in randomised order, also within blocks. The difference in torque levels was selected to be large enough to impact both intrinsic and reflexive pathways [14], while limiting fatigue. Moreover, in each block, the participant was motivated to find 5 different combinations of torque and depicted intrinsic stiffness (Block 1 & 3) or reflexive gain (Block 2 & 4). The participant was requested to hold each combination of torque and the impedance parameter (stiffness or reflexive gain) constant for 60 seconds, referred to as a ’hold period’. Between hold periods, participants searched for a new impedance parameter value different from the averages in previous hold periods.

The protocol was intended to measure a large range of intrinsic and reflexive impedances within each participant. This large range of variation is desired to properly investigate the association between the PC algorithm and EMG-based outcome measures. Participants could use the provided biofeedback to guide their modulation strategy across hold periods and to keep the parameters constant during the hold periods, see Fig. 1. No specific instruction on modulation strategies were given and co-contraction was permitted. Participants were instructed to keep away from the average impedance parameter magnitudes measured in previous hold periods. These average magnitudes were shown on screen as black-dashed vertical lines, see Fig. 1. Participants started the experiment with a screen without any black-dashed lines in Blocks 1 & 2 and placed an additional line with each completed hold period. The lines from Blocks 1 & 2 were used as starting point in Blocks 3 & 4 respectively.

### Online joint impedance estimation

**Fig. 2:**
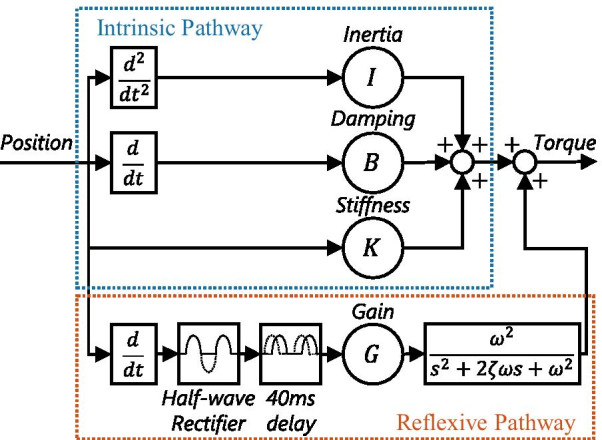
Parallel-cascade joint impedance model with intrinsic and reflexive pathway. The intrinsic pathway was modelled as a 2nd-order mass-spring-damper system with parameters: inertia *I*, damping *B* and stiffness *K*. The reflexive pathway was modelled based on the 40 ms delayed, half-wave rectified velocity using 2nd-order muscle activation dynamics and a parameter for reflexive gain *G*

The online algorithm of the PC system identification technique was used to simultaneously estimate intrinsic and reflexive impedances based on the model of Fig. 2. The PC model consisted of an intrinsic and reflexive pathway to relate the position perturbation as input with the measured torque response as output. The online PC algorithm required a 2$^{\circ }$ amplitude pulse-step position perturbation to be applied to the joint. Moreover, the PC model assumed a constant voluntary torque, therefore the initial impedance estimates after a change in torque target were unreliable for about 15 s [8, 9]

.

#### Algorithm

The implemented algorithm was based on the original algorithm combined with some specific improvements to decrease the bias on the identified parameters [8, 12]. The algorithm consists of the following 10 steps:

1. The measured position (with neutral position subtracted), velocity and torque signals were low-pass filtered (2nd-order, 100 Hz, Butterworth) to remove high-frequency noise.
2. The acceleration was estimated via numerical differentiation (4th order, backwards difference) of the low-pass filtered velocity.
3. The torque was high-pass filtered (2nd-order, 0.033 Hz, Butterworth) to remove any constant voluntary torque.
4. The 9 auto- and cross-correlation between position, velocity and acceleration, as well as the 3 cross-correlation between torque and position, velocity and acceleration were computed via a low-pass filter (2nd-order, 0.033 Hz, Butterworth).
5. The intrinsic inertia *I*, damping *B* and stiffness *K* parameters were estimated by solving an equation relating the 12 auto- and cross-correlations.
6. The intrinsic torque contribution was computed as defined in Fig. 2 using the estimated *I*, *B* and *K* parameters and high-pass filtered (2nd-order, 0.033 Hz, Butterworth) to remove the mean.
7. The reflexive torque was taken as measured torque minus intrinsic torque, see Fig. 2. The velocity was half-wave rectified and high-pass filtered (2nd-order, 0.033 Hz, Butterworth).
8. An anti-aliasing filter was applied to both reflexive torque and half-wave rectified velocity (8th-order, 81.9 Hz, 0.05 dB) and both were downsampled with a factor 10, to 204.8 Hz.
9. The reflexive IRF was estimated every 48.8 ms using a linear least-squares method, based on the reflexive torque and lagged half-wave rectified velocity (ranging from min. 50 ms to max. 400 ms lag) both with a data length of 1 s, see [8].
10. The reflexive gain *G* was computed as the sum of the reflexive IRF. The time series of reflexive gains *G* was then low-pass filtered (2nd-order, 0.033 Hz, Butterworth).

#### Pulse-step perturbation

The online PC algorithm required a purposely designed pulse-step position perturbation [8]. The estimation of the intrinsic parameters (Step 5) was based on the assumption that the cross-correlation between torque and position, velocity and acceleration are not affected by any reflexive contributions. The dedicated pulse-step perturbation signal was required to comply with this assumption and to avoid biased intrinsic parameter estimates. The signal randomly switched between ’pulses’, ramp-hold-return perturbations with a 40 ms width, and ’steps’, ramp-hold-return perturbations with a 460 ms width. The rising and falling edge position profiles were equal for pulses and steps and were generated by low-pass filtering (2nd-order, 30 Hz, critically-damped) a rate-limited (227.6 rad/s) block pulse. The perturbation was low-pass filtered and rate-limited to avoid excessive oscillations and overshoot in the imposed ankle position.

### Data analysis

The study outcome measures were based on the *K* and *G* parameters of the PC algorithm and the EMG measurements. For each hold period, an average of the intrinsic stiffness (*K*) and reflexive gain (*G*) was obtained. The model fit quality for each hold period was investigated by checking the amount of variance accounted for (VAF) of the measured torque ensemble. The torque ensemble was obtained by aligning all data at dorsiflexion perturbation onset and removing average background torque measured over the 40 ms period before onset. The online PC algorithm does not estimate all parameters of the PC model required to calculate model torque output, which is used to compute the VAF, see Fig. 2. Therefore, the unidentified activation dynamics parameters $\omega$ and $\zeta$ had to be estimated afterwards during data analysis. A nonlinear least squares optimization procedure was used per data point to find the $\omega$ and $\zeta$ maximizing VAF.

**Fig. 3:**
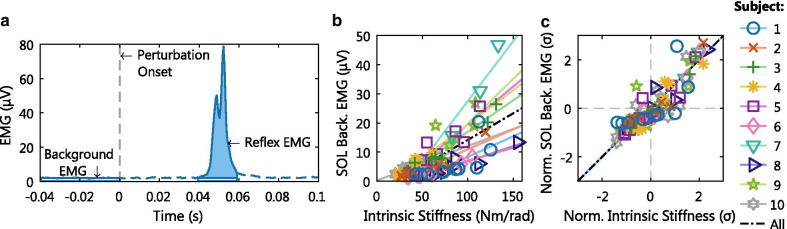
Data analysis methodology. **a** Background and reflexive EMG activity were calculated using the perturbation onset as reference. Background activity was based on the 40 ms period before perturbation onset, while reflexive activity was based on a 20 ms period about 40 ms after perturbation onset. **b** Absolute and **c** normalized correlation analysis. Both plots show a representative example using the intrinsic stiffness and SOL background EMG outcome measures collected at a 0 Nm torque target. A total least squares (TLS) fit shows the slope and intercept of the datasets of each individual participant

Average background and reflex EMG measures were calculated based on [10, 11], see Fig. 3a. Before analysis, the EMG measurements were high-pass filtered (2nd-order, 5 Hz, Butterworth) and rectified. The background EMG measure should reflect an average activity over the short, unperturbed period before perturbation onset. Therefore, background EMG activity was computed as the mean EMG activity over the 40 ms period before each dorsiflexion perturbation onset. The reflex EMG measure should reflect the true reflexive magnitude, observed as characteristic double-peak shape after rectification. Accordingly, reflex EMG activity was defined as the root mean square (RMS) of a subject-specific 20 ms window centered around M1 reflex activity. Before computing RMS, mean background activity was subtracted and the resulting signal was half-wave rectified. For the SOL, GM and GL reflex measures, dorsiflexion perturbation onset was used as timing reference. For the TA reflex measure, plantarflexion perturbation onsets of all steps were used as reference. Pulses were excluded for the TA, as the TA muscle stretch during a pulse follows only 40 ms after shortening.

Each hold period provided a paired data point between intrinsic stiffness and background EMG, and between reflexive gain and reflex EMG. A total of 200 hold periods (20 hold periods for 10 participants) were executed, equally split between the 0 and −5 Nm torque levels. Some data points were removed due to EMG measurement artifacts. Additionally, one data point was removed as the participant indicated that she had executed the task instructions incorrectly. She modulated the impedance parameter by deliberately varying voluntary torque. Therefore, 94 to 100 paired data points remained to investigate the linear associations.

Linear associations for both intrinsic and reflexive pathways were calculated on group-level using Pearson’s correlation coefficient, *r*. The correlation coefficient cannot be computed directly across the dataset, because the absolute values showed a subject-specific slope and intercept, see Fig. 3b. Therefore, all investigated datasets were normalized using the Z-score per participant. The Z-score standardization avoids any influence of subject-specific slopes and intercepts on the correlation coefficient, see Fig. 3c. The robustness of *r* was investigated using the 95% confidence interval (CI) constructed via a non-parametric bootstrap procedure using the bias corrected and accelerated method [18]. All data analysis was performed using Matlab 2017b (Mathworks, Natick, MA, USA).

## Results

We investigated the neurophysiological validity of an online PC algorithm, which disentangles the intrinsic and reflexive contribution to joint impedance. Participants completed 20 hold periods of 60 s with varying magnitudes of intrinsic and reflexive joint impedances across 2 voluntary torque levels, 0 and −5 Nm (plantarflexion). Each hold period provided a paired data point between estimated intrinsic stiffness and background EMG, and between estimated reflexive gain and reflex EMG. These paired data points were used to study the linear association by analyzing the correlation coefficient.

### Experiment time series

**Fig. 4:**
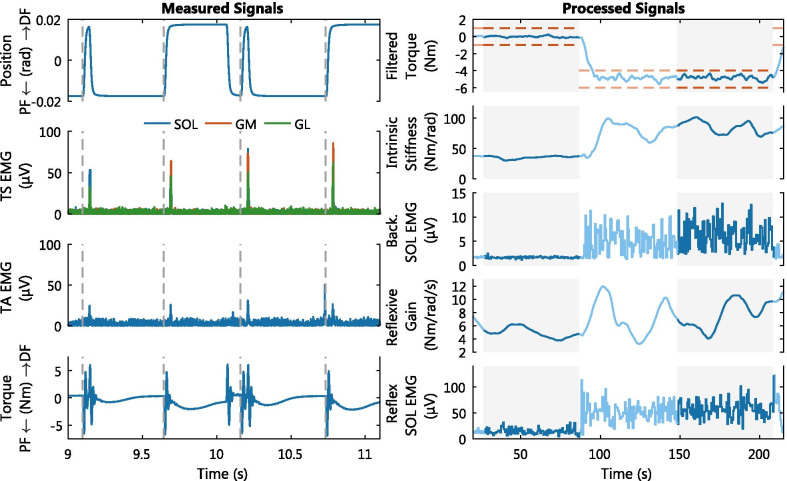
Time series of measured and processed signals, typical example for a single representative participant. (Left) Four consecutive dorsiflexion perturbations with perturbation onset (grey-dashed vertical lines). The response to the position perturbations are shown for the high-pass filtered, rectified EMG of Triceps Surae (TS) and TA as well as measured ankle joint torque. (Right) Two consecutive 60s hold periods (grey background) with transition period. 2D feedback was provided on torque and intrinsic stiffness. The time series show the voluntary modulation of the low-pass filtered torque and active torque target (red), intrinsic stiffness *K*, background SOL EMG activity, reflexive gain *G* and reflexive SOL EMG activity. The PC algorithm parameters *K* and *G* were computed continuously, while the EMG activity computations were performed around every perturbation onset

The measured time series signals show the stretch reflex elicitation and causality within the reflex loop in response to a position perturbation, see Fig. 4. The dorsiflexion perturbations stretch the ankle plantarflexors (SOL, GM and GL) and first show a reflexive EMG response after roughly 40 ms. This EMG response is followed by a contraction of the plantarflexors resulting in a reflexive plantarflexion torque with a peak roughly 150-200 ms after perturbation onset. Note, the antagonist TA muscle also appears to show reflexive EMG activity 40 ms after dorsiflexion perturbations, however this is considered to be cross-talk from the plantarflexors [9].

The processed time series show the simultaneous increase of the joint impedance parameters and EMG activity for both intrinsic and reflexive pathways across hold periods, see Fig. 4. The transition period between the hold periods lasted a minute, to have the participant familiarize themselves with the new task execution. Furthermore, the transition period is required to avoid violation of the online PC algorithm’s constant voluntary torque assumption.

### Hold period ensemble averages

**Fig. 5:**
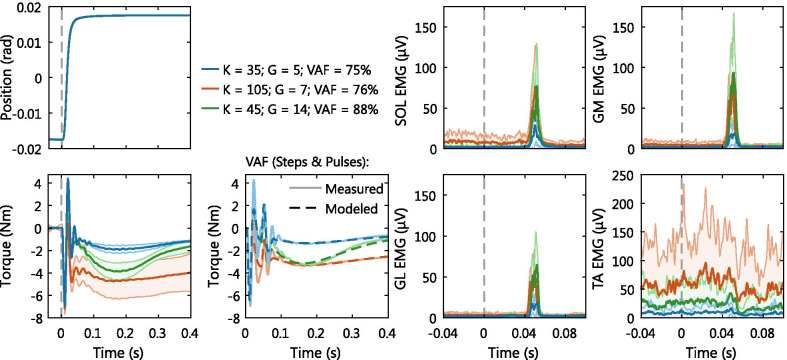
Ensemble averages (±SD) of hold periods with modulated impedance, typical examples for a single representative participant. Ensemble averages of the measured signals, created by aligning all step perturbations at perturbation onset (grey-dashed vertical lines). %VAF was computed using the measured and modeled torque ensemble of both step and pulse perturbations. The *K* (Nm/rad) and *G* (Nm/rad/s) parameter values provided represent the mean value across each hold period. All torque ensembles were normalized by subtracting the average background torque to enhance visualization of intrinsic and reflexive torque effects. All three hold periods were executed at a 0 Nm torque target

The simultaneous variation in joint impedance parameters and EMG activity was further investigated using the ensemble averages of each hold period, see Fig. 5. The model fitted the torque ensembles with a VAF of $76.2\pm 7.2\%$ with a range of $[56.9\ 88.6]\%$ across all hold periods and participants. The activation dynamics parameters found via nonlinear optimization to compute the VAF were: $\omega = 11.4\pm 2.0$ rad/s and $\zeta = 0.76\pm 0.12$. To check the constant voluntary torque assumption, the variance of the measured torque at all dorsiflexion perturbation onsets within a 60 s hold period was investigated. Across all participants, the hold periods showed a significantly lower torque average standard deviation at the 0 Nm target ($\sigma = 0.78\pm 0.26$) Nm than at −5 Nm ($\sigma = 1.32\pm 0.27$) Nm with $t(9) = -7.93$, $p =\ <.001$ (paired t-test).

An increased intrinsic stiffness was reflected in a larger plantarflexion torque across the 400 ms period after perturbation onset. Moreover, the increased stiffness was also reflected in increased background activity in both plantarflexors and dorsiflexors. This torque response matched with the concept of intrinsic stiffness, because the 2$^{\circ }$ dorsiflexion step perturbation lengthened the plantarflexor muscle-tendon unit over this entire 400 ms period. The EMG response showed the neural, non-velocity dependent contribution to joint impedance, with a large intrinsic stiffness matching high levels of co-contraction.

An increased reflexive gain was reflected in a larger reflexive plantarflexion torque with peak around 150-200 ms after perturbation onset. Moreover, the increased reflexive gain was also reflected in a larger EMG burst activity. The delayed timing with respect to perturbation onset and limited duration of both torque and EMG responses matches with the concept of a stretch reflex. The reflexive torque response is further delayed and smeared out compared with the EMG response due to the muscle activation dynamics, as included in the PC model Fig. 2. Note, EMG burst activity in the TA was observed in all participants after perturbations towards plantarflexion, stretching the TA, not the dorsiflexion perturbations shown in Fig. 5.

### Correlation analysis

**Fig. 6:**
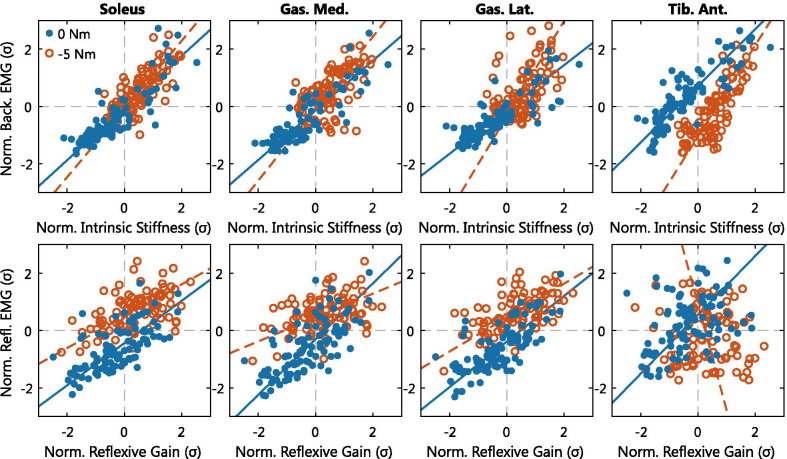
Linear associations between Z-score normalized joint impedance parameters and EMG activity across all participants. All datasets are shown for all hold periods across both torque levels. The TLS fit is shown for both torque levels to help visualize and interpret the linear associations

The consistency of the simultaneous variation of the joint impedance parameters and EMG activity across all participants and torque levels was investigated using Pearson’s correlation coefficient, see Fig. 6 and Table 1. For the intrinsic pathway, a positive correlation at both torque levels was observed for all muscles. For the reflexive pathway, a positive correlation at both torque levels was only observed for the plantarflexors.

The intrinsic pathway (top row of Fig. 6) showed fairly similar linear trends in the plantarflexors for the two torque levels, whereas the linear trend of the dorsiflexor differed between both torque levels. Furthermore, the correlation analysis at the −5 Nm level was restricted to smaller intervals for all muscles. All observations for the intrinsic pathway were caused by the additional plantarflexion activation required to reach a −5 Nm plantarflexion torque. On the other hand, the range of dorsiflexor muscle activity was not influenced by the −5 Nm level. These changes in muscle activation limit the range of plantarflexor activity and intrinsic stiffness. In contrast, maximum values for background EMG as well as intrinsic stiffness reached similar magnitudes at both torque levels.

**Table 1:** Pearson’s correlation coefficients (*r*) and their 95% confidence intervals across all hold periods *(N = 94-100)*

	Intrinsic		Reflexive	
Torque	0 Nm	−5 Nm	0 Nm	−5 Nm
SOL	0.89 [0.82, 0.93]	0.68 [0.53, 0.78]	0.64 [0.46, 0.75]	0.54 [0.34, 0.69]
GM	0.89 [0.82, 0.93]	0.54 [0.33, 0.67]	0.69 [0.57, 0.78]	0.31 [0.13, 0.50]
GL	0.87 [0.80, 0.92]	0.52 [0.35, 0.66]	0.68 [0.48, 0.78]	0.54 [0.38, 0.65]
TA	0.91 [0.84, 0.95]	0.84 [0.78, 0.89]	0.37 [0.13, 0.56]	$-0.02~[-0.24,0.20]$

Correlations between identified intrinsic stiffness and background EMG activity (intrinsic pathway) and identified reflexive gain and reflex EMG activity (reflexive pathway). The 95% confidence intervals were constructed using a non-parametric bootstrap procedure

The intrinsic pathway had a moderate to strong correlation with a range of $r=[0.52\ 0.91]$. If the two torque levels are considered separately, the degree of correlation was different between both levels for the three plantarflexors, based on the 95% CIs. The 0 Nm level showed a strong correlation ($r=[0.87\ 0.91]$) compared with a moderate correlation at −5 Nm ($r=[0.52\ 0.68]$). For the dorsiflexor, both torque levels showed a strong correlation ($r=0.91$ and 0.84).

The reflexive pathway (bottom row of Fig. 6) also showed fairly similar linear trends in the plantarflexors for the two torque levels, while the TA muscle trends differed. Again, low values of EMG activity were sporadically reached at the −5 Nm torque level, while maximum EMG values were more similar, especially for GM and GL. Contrary to the intrinsic pathway, the range of reflexive gain values did not seem restricted due to the −5 Nm torque level. Moreover, a relative shift appeared in the relation between EMG and reflexive gain in the lower range values (i.e. left hand side). The same level of reflex EMG corresponded to a lower level of reflexive gain in the −5 Nm task compared with the 0 Nm task.

The reflexive pathway had a weak to moderate correlation in the plantarflexors ($r=[0.31\ 0.69]$). The 0 Nm level showed a moderate correlation ($r=[0.64\ 0.69]$) compared with a weak to moderate correlation at −5 Nm ($r=[0.32\ 0.55]$). Contrary to the intrinsic pathway, the 95% CIs did overlap, except for the GM. The dorsiflexor had a weak correlation $r=0.38$ at 0 Nm and no correlation $r=-0.02$ at the −5 Nm level.

## Discussion

The main goal of this paper was to support the clinical application of the PC system identification technique through a neurophysiological validation on group-level. For the intrinsic pathway, a strong positive correlation between estimated intrinsic stiffness and background EMG was observed for plantarflexors and dorsiflexors at a 0 Nm voluntary torque level. For the reflexive pathway, a moderate positive correlation between estimated reflexive gain and reflex EMG was only observed for the plantarflexors at the 0 Nm torque level. For both intrinsic and reflexive pathways, a lower degree of correlation was found for the −5 Nm plantarflexion torque condition compared with a 0 Nm torque level.

### Linear association parallel-cascade system identification and EMG

The linear association between PC system identification technique and EMG outcome measures was previously only investigated for the reflexive pathway [9, 15, 16]. The multitude of outcome parameters used and the use of both between within- and between-subject measurements make it difficult to compare the previous results. Two studies investigated between-subject measurements. The first study investigated the intrinsic and reflexive ankle impedance components in stroke survivors using an offline PC algorithm [15]. The relative between-subject contribution of both intrinsic and reflexive impedance on the total response torque measured was investigated using the VAF. They found an unquantified positive association between the VAF by the reflexive contribution and reflexive EMG gain of GM or GL. The second study used an offline PC algorithm to investigate the intrinsic and reflexive contributions to wrist impedance in people with Parkinson’s disease [16]. The effect of medication on the neural, phasic component was studied by comparing the correlation between reflexive torque and reflexive EMG. For both on- and off-medication conditions moderate correlations of $r=0.45$ and 0.46 were found. Finally, one study investigated the within-subject voluntary modulation of reflexive impedance and stretch reflexes using the online PC algorithm [9]. For a single representative participant a correlation of $r \approx 0.98$ between reflexive gain and GL reflexive EMG was found to confirm that both measures modulated simultaneously.

The linear association between joint impedance and EMG is best investigated using within-subject measurements. Multiple subject-dependent characteristics influence both EMG amplitude, e.g. varying amounts of fat tissue, and joint impedance amplitudes, e.g. passive muscle slack length. These underlying subject-dependent characteristics would directly influence the linear association when between-subject measures are used. We applied a Z-score standardization to the data of each participant separately to compute a combined within-subject correlation coefficient. If all participants contribute the same number of samples, the combined correlation coefficient would equal the mean correlation of all ten participants. Therefore, our within-subject results can be compared directly with the within-subject results of [9].

Our results showed a lower correlation between reflexive gain and reflexive EMG of the GL than [9] at a 0 Nm torque target ($r = 0.68$ vs. 0.98). These results can potentially be explained by the differences in protocol and data analysis. First, the results of [9] were based on a single participant instead of ten. When calculating correlation coefficient for each individual a range of correlation values of $r=[0.04\ 0.94]$ was found compared with $r=0.68$ at group-level. However, as the individual results were based on only 10 paired data points per participant, a dedicated study design is recommended for validation on an individual level. Second, [9] did not allow co-contraction to reduce variation in intrinsic stiffness. This reduced variation could improve reflexive gain estimates, as simulation results showed that the online reflexive gain estimate is influenced by changes in intrinsic stiffness [8]. Third, the result of [9] was obtained in a second session, thus the participant was more familiar with the experiment and task. This familiarity could improve control over both reflexive impedance and torque and as result improve the quality of the parameter estimations.

The higher degree of correlation for the intrinsic compared with the reflexive pathway potentially reveals a better neurophysiological basis for the intrinsic pathway. However, the correlation sensitivity to both within-subject modulation range and amount of variation around the true value has to be taken as reservation. First, participants perceived modulating intrinsic impedance easier than reflexive impedance, as participants were used to conscious intrinsic stiffness modulation through co-contraction in daily living. This familiarity could have increased relative modulation range, thus resulting in larger correlations. Second, the reflexive gain parameter showed higher levels of variation during hold periods, see Fig. 4, which could result in a lower correlation. Note, the results of the dorsiflexor TA muscle was not taken into account when comparing correlations between the intrinsic and reflexive pathway. The dorsiflexor was excluded, because the input of the reflexive pathway in the PC model only uses the dorsiflexion perturbations, which stretch the plantarflexors, and not the plantarflexion perturbations, which stretch the dorsiflexors.

Despite the strong and moderate correlations found for the intrinsic and reflexive contributions to joint impedance, the neurophysiological basis of both pathways can be extended upon. Recent studies have shown that additional model parameters and elements are required to build complete models. The maximum VAF of 88.6% found in our results does indicate that there are unmodeled system dynamics in the experimental data. For the intrinsic pathway, a third-order model can better capture the agonist-antagonist musculoskeletal structure of the human ankle than the second-order IBK model used [19]. Fortunately, the estimated intrinsic stiffness component only shows a small bias for the 90-95$^{\circ }$ ankle positions used. For angles smaller than 90$^{\circ }$ the IBK model overestimates the stiffness, whereas for angles larger than 95$^{\circ }$ the IBK model underestimates the stiffness [19]. For the reflexive pathway, several studies about muscle spindles and spasticity have shown that the reflexive response is not only velocity dependent. Complete models would also potentially require elements based on acceleration, force and force derivative [20–22]. Note, all pulse and step perturbations applied during our experiment stretched the plantarflexors with exactly the same velocity, acceleration and force profile. Therefore, all observed modulations of joint impedance are attributed to task-driven changes made by the participants, which justifies the use of the PC model.

The 0 Nm torque target is recommended for future neurophysiological validation of joint impedance estimation algorithm, as it showed better characteristics for correlation analysis. Again, the sensitivity of the correlation analysis could explain the decrease in correlation at the −5 Nm target. First, Fig. 6 shows a smaller modulation range at −5 Nm on group-level, decreasing correlation. Second, correlation could have decreased due to increased variability as participants perceived it more difficult to keep torque constant at the −5 Nm level ($\sigma = 1.32$ Nm vs. 0.78 Nm at 0 Nm). The algorithm assumes this voluntary torque to be constant, thus torque variability can increase joint impedance estimation errors [8]. Third, small EMG magnitudes were occasionally measured from a specific muscle within a participant. As result, the amount of modulation observed also remained small. The small EMG magnitudes occurred most frequently within the GM or GL muscle in combination with the −5 Nm torque condition. These occurrences for the GM and GL are reflected in the lower group-level correlations compared to the SOL.

### Clinical application parallel-cascade system identification

The successful neurophysiological validation on group-level should support the clinical application of the PC model. This neurophysiological validation for the group of system identification methods is supported by the large degree of association between online and offline PC algorithms [8, 9]. A specific example of valid clinical applications would be within rehabilitation, utilizing the PC algorithms to unravel intrinsic and reflexive contributions. For example, this information could help in clinical decision making process to evaluate the current neurological impact of brain or neural injuries [6] or the effects of other treatments [7]. A strict limitation of the PC model is that isometric experimental conditions are required. Thus, self-generated movements cannot be analyzed using the PC model and other system identification techniques are required, e.g. [23]. Compared to our experimental conditions, recent advances did show that isometric conditions with faster variations in voluntary torques can be studied using the PC model [24]. As result, application of the PC model to evaluate functional tasks within a clinical setting, such as walking or balance, is difficult, because self-generated movement is a critical element of these tasks. Nevertheless, there is a relevant clinical need to unravel the contributions to joint hyper-resistance, even within an isometric context [2]. Still, the results do not show a perfect correlation between the joint impedance estimates and EMG measurements, which does raise a question which methodology is more suitable for use in clinical practise.

The lack of gold standard for reliably unravelling intrinsic and reflexive joint resistance contributions [2], makes it difficult to select the best method, EMG-based or PC-based, to quantify hyper-resistance in a clinical setting. Both methods could have their potential strengths and weaknesses depending on the hypothesized origin of a patients functional impairment and user aim. First, the joint impedance estimates and EMG measurements act at a joint and muscle level respectively. Second, the PC-based methods outcome measures are in mechanical units (*K* in Nm/rad and *G* in Nm$\cdot$s/rad), whereas EMG-based methods have electrical units of V. As result, the PC-based measures can be more directly related to the concept of resistance as felt by clinicians. Moreover, these outcome measures remove the need for normalization as required for the EMG-based methods to compare between-subject or across-session within-subject results. Third, the online PC algorithm showed slow variations, whereas online EMG measurements show fast variations, see Fig. 4. The online PC algorithm was purposely designed for these slow variations, as implementation of a 0.033 Hz low-pass filter improved participant control over the biofeedback [8]. Consequently, the online PC algorithm requires a transient period of about 15 s before estimates become reliable [8, 9]. On the other hand, due to the fast variations EMG-based methods are generally based on ensemble averages and hence require multiple perturbations to obtain a reliable measure as well. Fourth, PC-based methods require an experimental setup similar to Fig. 1 with a powerful actuator to apply the stretch perturbations. However, the need for EMG and sometimes even electrical stimulation [10, 11] equipment would be avoided. Contrarily, EMG-based methods can be used for motorized assessment, requiring less powerful actuators, and can even be executed without actuator at all via manual assessment [25].

### Study limitations

The study protocol design limits our results and conclusions to a group-level, as discussed above, and data periods of 60 s, assumed to have constant joint impedance. Moreover, the correlation coefficient used is sensitive to the ratio of within-subject modulation range and amount of variation around the true value. This effect influenced both the difference between the 0 and −5 Nm torque levels and the intrinsic and reflexive pathways. Furthermore, a limited amount of EMG activity in the GM and GL muscles in some participants also influenced the study outcome in a similar manner. To mitigate issues due to correlation sensitivity, biofeedback was provided on both torque and joint impedance with instruction to minimize variations within hold periods. Additionally, the joint impedance biofeedback helped to increase modulation range. Unfortunately, the large modulation range did in turn increase variability again for high intrinsic and reflexive impedance values, see Figs. 4 and 5. Moreover, the inclusion of several 60 s hold periods at high muscle activation levels also induce fatigue and hence again additional variability in the measurements. In short, participant instruction and protocol design were aimed to balance these multiple sources of variation to reduce their effect on the correlation coefficients.

## Conclusions

We have shown the neurophysiological validity of the PC system identification technique on group-level through the evaluation of an online PC algorithm. As hypothesized, for the intrinsic pathway, a strong positive correlation between estimated intrinsic stiffness and background EMG was found for both plantarflexors and dorsiflexors. For the reflexive pathway, a moderate positive correlation between estimated reflexive gain and reflex EMG was found for the plantarflexors only. For both intrinsic and reflexive pathways, a higher degree of correlation was found for the 0 Nm voluntary torque condition compared with a constant −5 Nm plantarflexion torque.

The successful neurophysiological validation shows the validity of the PC model and system identification techniques to study the human physiological system. The simultaneous validation of both intrinsic and reflexive pathways performed is important given the mix of physiological origins of joint hyper-resistance. As result, it is valid to use the PC system identification technique for the integrated assessment and training of participants with joint hyper-resistance in clinical practise.

## Data Availability

The code and data underlying this publication are available via 4TU. ResearchData: 10.4121/c.5281478.

## Abbreviations

CI: Confidence interval
EMG: Electromyography
GL: Gastrocnemius lateralis
GM: Gastrocnemius medialis
PC: Parallel-cascade
RMS: Root mean square
SOL: Soleus
TA: Tibialis anterior
TLS: Total least squares
TS: Triceps surae
VAF: Variance accounted for
